# Network meta-analysis of Chinese patent medicine adjuvant treatment of poststroke depression

**DOI:** 10.1097/MD.0000000000021375

**Published:** 2020-07-31

**Authors:** Ying Yu, Gong Zhang, Jing Liu, Tao Han, Hailiang Huang

**Affiliations:** a College of Traditional Chinese Medicine, Shandong University of Traditional Chinese Medicine; b Graduate Office of Shandong University of Traditional Chinese Medicine; c College of Rehabilitation Medicine, Shandong University of Traditional Chinese Medicine, Jinan, China.

**Keywords:** adjuvant therapy, Chinese patent medicine, network meta-analysis, poststroke depression, protocol

## Abstract

**Background:**

Poststroke depression is one of the common complications of clinical cerebrovascular diseases. It is commonly seen in 3 to 6 months after the onset of stroke. The incidence rate is 22% to 75%. The patient not only has depression-related emotional symptoms, but also are accompanied by autonomic nervous disorders and other physical symptoms. It will also delay the recovery time of patients’ neurological function, cognitive function, and limb function due to different degrees of depression, and even further aggravate the mortality and risk of accidental death of cerebrovascular disease. In recent years, Chinese patent medicine combined with western medicine has been widely used in the treatment of this disease. Many clinical practices have proved that the adjuvant treatment of pure Chinese herbal medicine can effectively alleviate the poststroke depression state and reduce the neurological deficits. The author has sorted out the relevant literature and data analysis to screen out the seven most representative and commonly used Chinese patent medicine preparations in clinical treatment of poststroke depression, which have certain clinical comparability when the dosage form and syndrome type are relatively unified. The network meta-analysis method is used to select the best clinical treatment plan, so as to provide reference value and evidence-based medicine evidence for the clinical optimization of drug selection.

**Methods:**

Using computer retrieval technology, comprehensive retrieval of CNKI, VIP, CBM, and WANFANG Chinese electronic database and the Cochrane Library, PubMed, Web of Science and EMBASE foreign electronic database. Search the clinical randomized controlled trials of these 7 kinds of Chinese patent medicines for adjuvant treatment of poststroke depression, and set a period of time is from the establishment of the database to May 31, 2020. The 3 authors will screen the literatures that meets the inclusion criteria, extract the data independently according to the predesigned rules, and evaluate the literature quality and bias risk of the included research according to the Cochrane 5.1 manual standard. R and the Aggregate Data Drug Information System software were used for data consolidation and network meta-analysis to evaluate the ranking probability of all interventions.

**Results:**

This network meta-analysis and probability ranking will identify the best Chinese patent medicine adjuvant treatment for poststroke depression.

**Conclusion:**

This study will provide systematic evidence-based medicine evidence for Chinese patent medicine adjuvant treatment for poststroke depression, and help clinicians, patients with poststroke depression and decision-makers to make more effective, safer, and economic optimal treatment plan in the decision-making process.

**PROSPERO registration number:**

CRD42020164543

## 1 Introduction

Poststroke depression is one of the common complications of cerebrovascular disease. It is commonly seen in 3 to 6 months after the onset of stroke, and the incidence rate is about 22% to 75%.^[1]^ Patients not only have emotional symptoms related to depression, but also are accompanied by autonomic nervous disorders and other physical symptoms. It will also delay the recovery time of patients’ neurological function, cognitive function and limb function due to different degrees of depression, and even further aggravate the mortality and risk of accidental death of cerebrovascular disease.^[2,3]^ Therefore, it is particularly important to effectively improve the clinical comprehensive efficacy, improve the neurological function and the quality of life of patients. At present, the traditional antidepressant drug are mostly used in western medicine. Although it can effectively improve the monoamine transmitter content in the synaptic space of patients and relieve the depression symptoms, along with the long-term medication cycle prolongation, the patients will have different degrees of adverse reactions and easy relapse after drug withdrawal, so it seriously restricts the patients’ treatment compliance and the curative effect after the disease.^[4,5]^

Traditional Chinese medicine, as an important part of complementary and alternative medicine, has played a huge advantage in the treatment of this disease. In recent years, Chinese patent medicine combined with western medicine has been widely used in the treatment of this disease. It can not only can effectively avoid the addiction, toxicity, drug resistance and other deficiencies caused by the long-term use of western medicine, but also can show the characteristics of the overall regulation of the viscera of TCM, and then it can quickly alleviate the depression, shorten the rehabilitation time and improve the quality of life. Based on the relevant literature and data analysis to screen out 7 kinds of the most representative and commonly used oral Chinese patent medicine in treatment of poststroke depression, so it has certain clinical comparability under the condition that the dosage form and syndrome type are relatively unified. However, most of the current studies only report the efficacy of oral Chinese patent medicine in the treatment of poststroke depression by conventional paired meta-analysis, but there is no evidence-based evaluation of the clinical efficacy and safety of the 7 kinds of Chinese patent medicine in the treatment of poststroke depression. Therefore, the purpose of this study is to use the network meta-analysis (NMA) method to integrate the clinical relevant evidence of direct and indirect comparative relationship, to analyze and sort the quantitative data of different Chinese patent medicines that treat the same evidence body of the disease, and then the best clinical treatment scheme was selected, so as to provide reference value and evidence-based medical evidence for the clinical optimization of drug selection.

## 2 Methods

### 2.1 Protocol registration

A report on the further results of this study will be submitted in accordance with the guidelines of the PRISMA NMA extension statement.^[6]^ In April 2020, we have obtained the registration number (CRD42020164543) of this study on the platform of PROSPERO (https://www.crd.york.ac.uk/prospero/). Because the NMA protocol has been approved by the local agency review committee and the ethics committee, it does not involve privacy information and does not require further ethical approval and informed consent.

### 2.2 Information sources

By using the method of computer retrieval technology, the clinical randomized controlled trials (RCTs) of 7 kinds of oral Chinese patent medicine adjuvant treatment of poststroke depression were searched. The primary search was selected and set a period of time is from the establishment of the database to May 31, 2020. The computer retrieval electronic database included CNKI, CBM, WANFANG data, VIP, and other Chinese databases, as well as the Cochrane Library, PubMed, Web of Science, and EMBASE and other foreign databases. Search terms include: Chinese patent medicine, Shuganjieyu capsule, Wuling capsule, Yangxueqingnao granule, Chaihushugan powder, Jieyuanshen capsule, Xiaoyao pills, Danzhixiaoyao pills, Poststroke depression, Depression syndrome after stroke, Randomized controlled trials. Different databases choose the corresponding combination of subject words, free words and keywords.

In addition, the relevant journals shall be searched in the reference literature, and the relevant literature shall be tracked, and Google scholars shall be used together Baidu academic and other relevant search engines conduct relevant research on the Internet by hand, and will provide data for all relevant authors and major researchers to supplement the incomplete report or unpublished research of the original paper. We will try our best to ensure that the primary search work is comprehensive so as not to lose valuable research materials. In Table 1, the preliminary search strategy of PubMed database is taken as an example to summarize the preliminary search strategy, which will be adjusted according to the requirements of other electronic databases related to keywords.

**Table 1:**
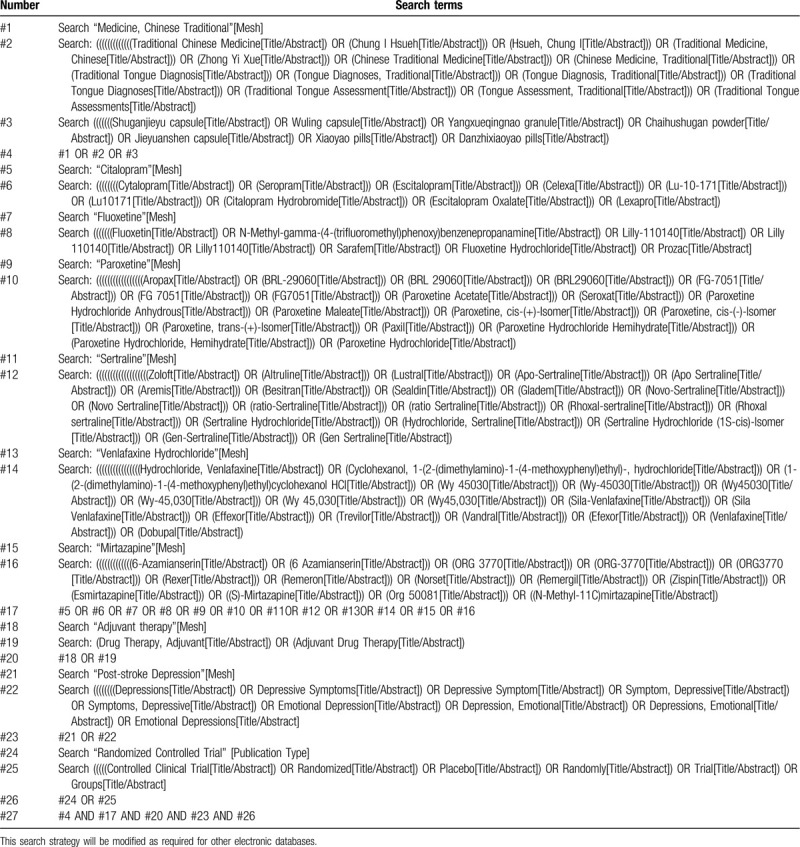
Search strategy used in PubMed database.

### 2.3 Eligibility criteria

The design of inclusion criteria and exclusion criteria in this study is based on the 5 main principles of PICOS (Participant-Intervention-Comparator-Outcomes-Study design).

#### 2.3.1 Type of participant

The patients were single poststroke depression patients, and their age, gender, and race were unlimited. The diagnosis standard for post-stroke depression refers to the “Guidelines for the Diagnosis and Treatment of Acute Ischemic Stroke in China” (2014 version) revised by the Cerebrovascular Diseases Group of the Neurological Branch of the Chinese Medical Association in 2014, the onset of stroke is in the recovery period or sequelae period.^[7]^ The diagnosis standard for depression refers to the “Classification and Diagnosis Criteria of Chinese Mental Disorders” (Third Edition).^[8]^ The diagnostic standard of traditional Chinese medicine refers to the diagnostic standard of stroke and depression in the diagnostic efficacy standard of traditional Chinese medicine.^[9]^

#### 2.3.2 Type of interventions and comparators

In the case of clear diagnosis standard, curative effect judgment standard and consistent basic treatment, the treatment group was treated with Chinese patent medicine (Shugan Jieyu capsule or Wuling capsule or Yangxueqingnao granule or ChaiHuShuGan powder or Jieyu Anshen capsule or Xiaoyao Pill or Danzhi Xiaoyao Pill) combined with western medicine (Citalopram or Fluoxetine or Paroxetine or Sertraline or Venlafaxine Hydrochloride or Mirtazapine), and the control group was treated with western medicine (Citalopram or Fluoxetine or Paroxetine or Sertraline or Venlafaxine Hydrochloride or Mirtazapine).

#### 2.3.3 Type of outcomes

The predetermined evaluation outcome measures mainly include:

1. clinical efficacy,
2. Hamilton Depression Scale (HAMD) score,
3. Treatment Emergent Symptom Scale (TESS) score,
4. National Institute of Health Stroke Scale (NIHSS) score.

#### 2.3.4 Type of study

The literature included were RCTs with no limitation on language and blind or assignment concealment. In addition, the author will exclude the non RCTs, case report, experience summary, self-control and review literature, animal experimental research, and repeatedly published literature. At the same time, the diagnosis of poststroke depression is not clear or literature combined with other diseases, the efficacy judgment standard of the test group and the control group is not clear, and the treatment measures involve other treatment and affect the final treatment of causality judgment literature, literature with unclear research results, incomplete data or no connection with the full text author is also excluded.

### 2.4 Study selection and data extraction

**Figure 1:**
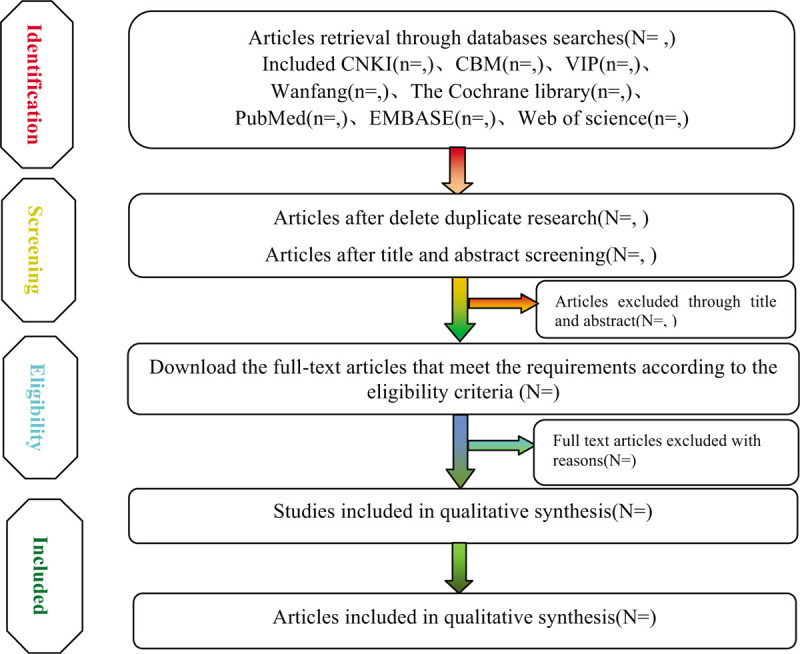
Flow diagram of study.

According to the retrieval strategy of the above electronic databases, three researchers searched the electronic databases, used EndnoteX9 software to search the repeated information, combined the literature retrieval results in different databases, established the information database and downloaded the full text. Then, 2 researchers independently conducted the data extraction according to the preformulated form, and took cross-checking and review. The process of study selection will be summarized in the PRISMA flowchart in Figure 1.^[10,11]^

Data extraction content includes

1. Basic information of the included literature (including the first author, published journal and year, research topic).
2. Relevant information of the treatment group and the control group in the literature (including the number of cases, total cases, age, intervention measures, course of treatment, outcome indicators).
3. Design type and quality evaluation information of the included literature.
4. Outcome measures (Clinical efficacy, HAMD score; TESS score; NIHSS score.)

### 2.5 Study quality evaluation

According to the quality evaluation standard of Cochrane system evaluator manual, Revman quality evaluation tool was used to evaluate the methodological quality of the included study, including random method, assignment concealment, blind method, outcome data integrity, selective report, number of dropped cases, follow-up and other biases. Each project was divided into 3 options: high risk, low risk, and uncertainty risk, according to the description of the above aspects in the included research, the 2 first authors independently completed the quality evaluation results of the included literature. If there are differences in the results, it is necessary to invite the third researcher to help each other discuss and make interpretation and quality evaluation. According to the standards of Cochrane manual, literature quality assessment and bias risk assessment were carried out. R software and the Aggregate Data Drug Information System (ADDIS) software are used for statistical software, data integration and NMA.^[12,13]^

Consulting GRADE handbook, the assessment which would be carried out through the Grading of Recommendations Assessment, Development, and Evaluation (GRADE, https://gradepro.org/)^[14]^ by 2 independent authors will be designated into 4 grades: high quality, moderate quality, low quality, and very low quality.

### 2.6 Data synthesis and statistical methods

#### 2.6.1 Pairwise and network meta-analysis

Revman software is provided by Cochrane collaborative network for literature quality and bias risk assessment. R language programming and ADDIS software is used for direct and indirect results comparison and 95% confidence interval (CI) calculation in network meta-analysis. At the same time, network relationship diagram and anecdotal sequence diagram of various interventions are drawn, which can effectively show the indirect comparison relationship of 7 kinds of oral Chinese patent medicine. The network diagram is mainly composed of nodes and line composition, in which node represents a treatment mode, the node connected by a straight line represents a direct or indirect comparative relationship between the 2, and the thickness of the connecting line represents the number of studies. Then, we will analyze the results of all direct or indirect comparisons to evaluate which is the best treatment plan for poststroke depression by these 7 kinds of Chinese patent medicine, and estimate the rank probability of each group based on Markov chain Monte Carlo method. R programming language starts “NETMETA” program, and calls Bayes Markov China Monte Carlo algorithm to analyze the data of random effect model.^[15]^ The odd ratio value was used as the statistical value of efficacy analysis, the weighted mean difference or standardized mean difference was used as the measurement data, and 95% CI was used for each effect. Odd ratio was used as the statistical measure of effective rate, healing time of related symptoms and adverse reactions, and 95% CI confidence intervals was used to express the effect. Based on the network meta probability ranking, the larger the probability *P*-value of clinical total effective rate, the better. The smaller the *P*-value of HAMD, TESS, and NIHSS score, the better. ADDIS software uses related instructions to call the data results of the random effect model based on Bayesian Markov China Monte Carlo algorithm for prior evaluation and processing (4 chains are used for simulation analysis, the initial value is 2.5, the iteration step is refined by 10, the number of iterations is adjusted by 20,000, and the number of simulation iterations is 50,000).^[16]^

#### 2.6.2 Assessment of heterogeneity

Heterogeneity will be evaluated by Cochrane. For each pairing comparison, statistical heterogeneity will be evaluated by *I*^*2*^ index, subgroup analysis based on the heterogeneity factors and study by χ^2^ test. Evaluate the clinical and method heterogeneity of the included study, and compare the fitting degree of fixed effect model and random effect model. If each study in the subgroup has statistical homogeneity (*P* ≥ .1, *I*^*2*^ ≤ 50%), the fixed effect mode is used for meta-analysis. Otherwise, the causes of heterogeneity are analyzed first, and the random effect mode is used for meta-analysis without obvious clinical heterogeneity (*P* < .1, *I*^*2*^ > 50%), and the possible causes of heterogeneity are found out from both clinical and methodological aspects. If the clinical trial data provided cannot be meta analyzed, descriptive analysis shall be conducted.

#### 2.6.3 Subgroup and sensitivity analyses

If the result of meta-analysis is positive and there are more than 3 included studies, R software shall be used to conduct sensitivity analysis on the statistical results, and meta-analysis shall be carried out again for each excluded study, and the results shall be compared with those before exclusion. If there is no substantial change in the comparative analysis, the results are stable. Otherwise, the data results are not stable. If significant heterogeneity is found, subgroup analysis will be envisaged based on treatment time, age, race, gender, and quality of study to investigate possible sources of heterogeneity.

#### 2.6.4 Assessment of inconsistency

The Node-Split Model of ADDIS software is used to test the inconsistency. If there is no statistical difference (*P* > .05) in each study within the subgroup, it indicates that the heterogeneity of the included study is small, so the consistency model is used for analysis. Otherwise, the Inconsistency Model is used for analysis. Potential scale reduced factor reflects convergence. When potential scale reduced factor is close to 1 or equal to 1, it indicates that it has achieved better convergence efficiency and the results of consistency model analysis are reliable.

#### 2.6.5 Publication bias

If more than 5 studies are included, R software is used to analyze the potential publication bias, and the figure is inverted funnel-shaped and symmetrical, which indicates that the possibility of publication bias is relatively small. If the figures are biased, it indicates that there is a greater possibility of publication bias.

## 3 Discussion

Traditional Chinese medicine often adopts the treatment methods of soothing the liver and relieving depression, promoting qi and dredging collaterals, nourishing yin and blood, activating blood circulation and removing stasis, which can not only give full play to the advantages of multiple targets and multiple pathways among herbal medicines, but also stimulate the synergistic effect of active ingredients among herbs to produce antidepressants. This study screened out seven kinds of commonly used oral Chinese patent medicines, namely Shugan Jieyu capsule, Wuling capsule, Yangxueqingnao granule, ChaiHuShuGan powder, Jieyu Anshen capsule, Xiaoyao Pill and Danzhi Xiaoyao Pill, which were all made from the combination of pure natural Chinese herbal medicines. Meanwhile, traditional Chinese medicine has mild properties, so it can effectively reduce the number of clinical adverse reactions on the basis of definite efficacy, so as to greatly improves patient's compliance and tolerance. In terms of dosage form, Chinese patent medicine cannot only avoid many inconveniences caused by herbal decoction, but also make use of sucrose auxiliary materials to adjust the taste, so that it is very easy to be accepted by patients in clinical. In addition, its clinical convenience, standardization, safety, and other aspects continue to improve, which greatly promotes the clinical cure rate, shortening the course of disease, improving the safety and compliance of clinical treatment. Therefore, it has a certain clinical application value.

Under the condition that the syndrome types and oral dosage forms of traditional Chinese medicine are relatively consistent, the direct intervention treatment of 7 kinds of Chinese patent medicine have certain clinical comparability. However, there is no standardized diagnostic standard and therapeutic effect judgment standard for treatment of poststroke depression disease by traditional Chinese medicine. At present, most of studies are focus on the clinical efficacy of the 2 comparative studies of traditional Chinese medicine oral liquid in the treatment of poststroke depression disease, and there is still a lack of network meta-analysis on the clinical efficacy and safety to compare multiple traditional Chinese patent medicine in the treatment of this disease in the same clinical trial. Therefore, the purpose of this study is to use a high-quality system to evaluate the commonly used Chinese patent medicines, and to use the NMA method to obtain the analysis of the clinical efficiency, HAMD, TESS, and NIHSS score of the seven kinds of Chinese patent medicine adjuvant treatment, so as to determine the antidepressant effect of the 7 kinds of Chinese patent medicines, and then rank the probability according to the advantages and disadvantages of the index effect. Then, the best evidence of clinical treatment measures will be screened out, and the quality of the evidence will be evaluated by the hierarchical method. Through a comprehensive analysis of the data, it can be said that it has a certain reference value for clinicians to select the best Chinese patent medicine. However, there are certain defects in our NMA, such as publication bias, clinical heterogeneity, and selection bias, which will ultimately affect the recognition of the research results. While we still hope that this study can provide the best possible drug selection and reliable evidence-based medicine for clinical practice, and to some extent provide strong evidence for the significant advantages of traditional Chinese patent medicine in the treatment of poststroke depression, so it can provide more reliable reference value for clinical practice.

At present, the protocol for the NMA has been registered on the international system review expectation register (CRD42020164543), which will follow the guidelines of “Cochrane Intervention System Review Manual” and “ Preferred Reporting Items for Systematic Reviews and Meta Analyses Protocols statement.” In addition, if the protocol needs to be amended, there will be a description of the amendment with the reason and the date.

## Author contributions

**Conceptualization:** Ying Yu, Gong Zhang and Jing Liu.

**Project administration:** Tao Han, Hailiang Huang.

**Data curation:** Ying Yu, Gong Zhang and Jing Liu.

**Formal analysis:** Ying Yu, Gong Zhang and Jing Liu.

**Methodology:** Ying Yu, Gong Zhang and Jing Liu.

**Software:** Ying Yu, Gong Zhang and Jing Liu.

**Supervision:** Tao Han, Hailiang Huang.

**Writing – original draft:** Ying Yu, Gong Zhang and Jing Liu.

**Writing – review & editing:** Ying Yu, Gong Zhang and Jing Liu.

## References

[1] Huf W Steckel R Sitezer M Poststmke depression: risk factors and effects on the course of the stroke. Nervenzrzt 2003;74:104–14. 10.1007/s00115-002-1417-x 12596011

[2] Sivolap YP Damulin IV Stroke and depression. Zh Nevrol Psikhiatr Im S S Korsakova 2019;119:143–7. 10.17116/jnevro2019119091143 31626232

[3] Paolucci S Epidemiology and treatment of post-stroke depression. Neuropsychiatr Dis Treat 2008;4:145–54. 18728805 10.2147/ndt.s2017 PMC2515899

[4] Loubinoux I Kronenberg G Endres M Post-stroke depression: mechanisms, translation and therapy. J Cell Mol Med 2012;16:1961–9. 22348642 10.1111/j.1582-4934.2012.01555.x PMC3822966

[5] Shelton RC Osuntokun O Heinloth AN Therapeutic options for treatment-resistant depression. CNS Drugs 2010;24:131–61. 20088620 10.2165/11530280-000000000-00000

[6] Shamseer L Moher D Clarke Mike Preferred reporting items for systematic review and meta-analysis protocols (PRISMA-P) 2015: elaboration and explanation. BMJ 2015;1:350. 10.1136/bmj.g7647 25555855

[7] Neurology branch of Chinese Medical Association, cerebrovascular disease group of Neurology branch of Chinese Medical Association. Guidelines for the diagnosis and treatment of acute ischemic stroke 2014. Chin J Neurol 2015;48:246–57.

[8] Chinese Medical Association Psychological Science Society. Classification and diagnostic criteria of mental disorders in China (Third Edition). Chin J Psychiatry 2001;34:184–8.

[9] State Administration of traditional Chinese medicine. Diagnostic efficacy standard of traditional Chinese medicine disease. 2012;Beijing: China Medical Science and Technology Press, 33–39.

[10] Hutton B Salanti G Caldwell DM The PRISMA extension statement for reporting of systematic reviews incorporating network meta-analyses of health care interventions: checklist and explanations. Ann Intern Med 2015;162:777–84. 26030634 10.7326/M14-2385

[11] Liberati A Altman DG Tetzlaff J The PRISMA statement for reporting systematic reviews and meta-analyses of studies that evaluate health care interventions: explanation and elaboration. J Clin Epidemiol 2009;62:1–34. 19631507 10.1016/j.jclinepi.2009.06.006

[12] Hongqiu G Yang W Wei L Application of Cochrane bias risk assessment tool in meta-analysis of randomized controlled study. China Circ J 2014;29:147–8.

[13] Jie M Ying L Laiping Z Application and comparison of Jadad scale and Cochrane bias risk assessment tool in quality evaluation of randomized controlled trials. Chin J Oral Maxillofac Surg 2012;10:417–22.

[14] Guyatt G Oxman AD Ak EA GRADE guidelines: 1. Introduction-GRADE evidence profiles and summary of findings tables. J Clin Epidemiol 2011;64:383–94. 21195583 10.1016/j.jclinepi.2010.04.026

[15] Chao Z Feng S Xiantao Z R software calls JAGS software to realize network meta analysis. Chin J Evid Based Med 2014;14:241–8.

[16] Van Valkenhoef G Tervonen T Zwinkels T ADDIS: a decision support system for evidence-based medicine. Decision Support Systems 2013;55:459–75.

